# Age-associated microglial heterogeneity includes emergence of mobile microglial states

**DOI:** 10.3389/fnagi.2026.1847223

**Published:** 2026-06-30

**Authors:** Sunitha Subhramanian, Olga Bocharova, Olga Mychko, Natallia Makarava, Ilia V. Baskakov

**Affiliations:** Department of Neurobiology, University of Maryland School of Medicine, Baltimore, MD, United States

**Keywords:** aging, calcium, microglia, microglial surveillance, mobility, P2y12, prion diseases, time-lapse imaging

## Abstract

Microglia maintain neuronal homeostasis through dynamic surveillance strategies that depend on their functional state. In the healthy brain, highly ramified microglia monitor neuronal integrity via motile processes and transient soma contacts. Aging is associated with reduced process motility and diminished expression of homeostatic markers, raising the question of how microglial surveillance adapts to these changes. Here, we used *ex vivo* time-lapse imaging of acute cortical slices from young and aged mice to characterize age-dependent alterations in microglial behavior. We found that, unlike microglia in young animals, a subset of aged microglia exhibited pronounced somatic mobility and loss of territorial confinement, resembling migratory behaviors previously described in neurodegenerative conditions. Unsupervised clustering analyses revealed increased heterogeneity in aged microglia, including the emergence of distinct high-mobility subpopulations absent in young brains. Despite these dynamic changes, aged microglia largely retained ramified morphology and did not show increased neuronal envelopment. In parallel, microglia from aged mice displayed prolonged intracellular Ca^2+^ bursts across all subpopulations, indicating a global shift in functional state. These sustained calcium signals resembled those observed in disease-associated microglia, suggesting that aging induces a primed, partially activated phenotype. Consistent with this interpretation, we observed a trend toward reduced P2Y12 expression in aged microglia. Together, our findings demonstrate that microglia adapt to aging by shifting from process-based surveillance toward increased soma-mediated mobility, accompanied by sustained Ca^2+^ signaling. This adaptive response may compensate for declining process dynamics while reflecting a transition toward a pro-inflammatory, disease-associated state.

## Introduction

Microglia are the resident immune cells of the central nervous system and play a central role in monitoring neuronal activity and integrity. To survey neuronal status, microglia adopt distinct strategies depending on their functional state. In the healthy brain, homeostatic microglia exhibit a highly ramified morphology and dynamic process behavior, characterized by continuous extension and retraction - a phenomenon termed microglial motility (Damani et al., 2011; Rotterman and Alvarez, 2020; Cserép et al., 2022; Nebeling et al., 2023). These processes form direct contacts with neuronal somata, establishing specialized structures known as somatic purinergic junctions (Cserép et al., 2020). Such transient process-to-soma interactions constitute a primary mechanism of neuronal surveillance under homeostatic conditions.

In chronic neurodegenerative states, microglia acquire a sustained reactive phenotype (Keren-Shaul et al., 2017; Krasemann et al., 2017; Butovsky and Weiner, 2018; Makarava et al., 2020). Reactive microglia differ markedly from their homeostatic counterparts, adopting an amoeboid morphology with reduced process complexity. Recent studies in prion-infected mice have demonstrated that reactive microglia exhibit increased somatic mobility, actively migrating through the brain parenchyma and forming direct, transient, and often extensive cell body-to-cell body contacts with neurons (Subhramanian et al., 2026). These interactions frequently involve partial or complete somatic envelopment and can persist from minutes to several hours, with dynamic and reversible transitions between states (Makarava et al., 2024). Notably, neuronal envelopment by reactive microglia has been observed in both murine prion disease and human sporadic Creutzfeldt–Jakob disease (sCJD) (Makarava et al., 2024), suggesting a conserved biological mechanism.

Aging is also associated with significant alterations in microglial phenotype. With advancing age, microglia display reduced ramification and increased expression of pro-inflammatory cytokines (Sierra et al., 2007; Hefendehl et al., 2014; Hammond et al., 2019). Transcriptomic analyses have identified a subset of microglia in the aged brain that exhibit a chemokine-enriched, pro-inflammatory profile resembling disease-associated microglia (DAM) described in multiple neurodegenerative conditions (Holtman et al., 2015; Olah et al., 2018; Hammond et al., 2019; Sankowski et al., 2019). Concomitantly, age-related reductions in process number and length diminish the territorial coverage of individual microglial cells, leading to impaired surveillance of neuronal populations (Hefendehl et al., 2014). In addition, the responsiveness of microglial processes, particularly their rate of extension toward focal injury, is significantly attenuated with age (Hefendehl et al., 2014). Together, these changes pose a fundamental challenge: as the demand for neuronal surveillance increases with aging, the efficiency of process-based monitoring declines. How microglia adapt their surveillance strategies to maintain homeostasis in the aging brain remains unclear.

To address this question, we investigated microglial surveillance behavior during normal aging using *ex vivo* time-lapse imaging of acute brain slices. Unexpectedly, in contrast to microglia from young animals, a subset of microglia in aged mice exhibited loss of territorial confinement and increased somatic mobility, reminiscent of reactive microglia observed in prion-infected mice. Furthermore, microglia in aged brain slices displayed prolonged and sustained Ca^2+^ transients. These findings suggest that, with normal aging, microglia undergo a shift in surveillance strategy, potentially compensating for diminished process-mediated monitoring.

## Materials and methods

### Animals

Two age groups of B6.129P2(Cg)-*Cx3cr1*^*TM*1*Litt*^/J mice abbreviated as Cx3cr1/EGFP (Strain 005582, The Jackson Laboratory) were used for preparing acute brain slices: young adult: 46–55-days-old mice (1M/2F), and aged: 605–607 days old mice (1M/2F). For prion-infected mice, Cx3cr1/EGFP mice were inoculated via intraperitoneal route with 200 μl of 1% SSLOW brain homogenate as previously described (Makarava et al., 2021), and brain slices were analyzed at clinical stage of the disease.

### Antibodies

Primary antibodies used for immunofluorescence: goat polyclonal anti-IBA1 (#NB100-1028, Novus, Centennial, CO); mouse monoclonal anti-NeuN, clone A60 (#MAB377, Millipore Sigma, Burlington, MA); rabbit polyclonal anti-P2Y12 (#55043A, Anaspec, Fremont, CA). The secondary antibodies for immunofluorescence were Alexa Fluor 488-, 546-, and 647-labeled (ThermoFisher Scientific, Waltham, MA).

### Acute brain slice preparation and *ex vivo* time-lapse imaging

Before euthanizing an animal, sterile PTFE hydrophilic membrane inserts (PICM0RG50, Sigma) of 0.4 μm pore size were kept in a 6-well plate supplemented with serum free culture media and incubated at 37 °C and 5% CO_2_ for 1–2 h in a standard cell culture incubator. For acute slice preparation, the whole mouse brain was removed from the skull and were immersed in ice cold oxygenated ACSF media (LRE-S-LSG10001, Ecocyte Bioscience LLC) saturated with 95% O_2_ and 5% CO_2_. The cerebellum and olfactory bulb were cut off and the remaining portion of the brain was glued to the bottom of the specimen holder within the tissue slicing chamber of the Vibratome (Leica VT1200) filled with oxygenated ACSF media such that the ventral part was facing toward us. Acute coronal cortical sections of 20 μm thickness were prepared using the vibratome at 4.5 mm amplitude, 84–86 Hz frequency and 2.0–2.5 mm/s speed. Next, the membrane inserts were incubated with fresh complete growth medium containing 50% MEM (INV-42360032, Invitrogen), 25% BME (INV-21010046, Invitrogen), 5% heat inactivated horse serum (INV-26050070, Invitrogen), 10 ng/ml nerve growth factor (NGF) (INV-A42627, Invitrogen) and GDNF (INV-AF-450-44, Invitrogen). The sections were transferred using a sterile pasteur pipette to the inserts and stained with 0.5 mkM Hoechst 33342 (INV-H3570, Invitrogen) for 10 min followed by washing in ACSF media. The slices were then transferred to coverslip bottom petri dish (VWR-MSPP-P35G014C-CS, Mattek Corp MS) for their time-lapse imaging in Leica MICA Widefield Live Cell microscope (Leica Microsystems, Deerfield, IL). For Ca^2+^ imaging, slices were incubated simultaneously with 0.5 mkM Hoechst and 0.5 mkM Calbryte 590 AM (VWR-76484-390-EA, AAT Bioquest) for 45 min.

### Time-lapse imaging

The time-lapse imaging were performed using environmental climate setup in Leica MICA supplied with 5% CO_2_ at 37 °C to ensure slice viability. Image acquisition parameters for all channels (EGFP, Hoechst and Calbryte 590AM) were kept at 100 ms exposure, with the constant focusing on the green channel (EGFP) to avoid any focal drift. Images were captured by the 10× objective every 3 min for 3 h. Background for the videos were deduced using the thunder and lightning module.

### Microglial cell tracking and analysis

Time-lapse videos were loaded into ImageJ (FIJI). Using Manual tracking and MtrackJ plugin, total distance was calculated as the sum of the incremental distance measured from all frames, while track displacement was calculated as the change in position from the first to the last frame. Mean speed was calculated as the average of all velocities linking the various tracks. The Rose plots were build using Chemotaxis tool software (Ibidi GmbH, Germany).

### UMAP and PC clustering

Time-lapse recordings were analyzed using TrackMate (FIJI/ImageJ) for generating TrackMate XML files comprising of the trajectory/track datasets. Tracking was performed for each EGFP^+^ cell within the 500 × 500-pixel frames. TrackMate XML files were exported into an open-source statistical analysis platform R (R Foundation for Statistical Computing^1^) using CellRomeR: import_XML for the subsequent downstream analysis.

Clustering and multivariate analysis were performed in R by using the following packages: CellRomeR (TrackMate XML import), data.table (data handling), uwot (UMAP), ggplot2 (plotting), RColorBrewer (color palettes) and scales (plot utilities). The track tables in TrackMate XML files were exported into a data.table. To maintain provenience, each cell from the trackmate import files was treated as a.sample identifier and recorded with a unique Sample_ID. Then, for each track, we constructed a featuring matrix constituting the following TrackMate-derived mobility parameters available in the XML files: Track Mean Speed, Track Maximum Speed, Track Minimum Speed, Track Median Speed, Track Std Speed, Linearity of forward progression, Track Displacement, Mean Straight Line Speed, Total Distance Traveled, Max distance traveled and Mean directional change rate. Columns were then complemented robustly by case-insensitive keywords, and transformed into numeric for multivariate analyses.

Before clustering, the track-level mobility parameters were standardized by z-score values. Then, UMAP features was computed using the scale feature matrix using uwot package, providing fixed values for reproducibility among sample groups. Unsupervised clustering was then performed on the same scaled feature matrix using k-means with automatically generated clusters (k). Cluster label was stored as a factor and then merged back to the UMAP coordinates linking the original track features. The following output files were generated: (i) UMAP coordinate track table, and (ii) a cluster-level mean feature values summary table. For refining the UMAP data points with a tighter cluster boundary, outliers were removed within each cluster using a median approach deviation (MAD) method, which was applied to the radial distance of each data point from the cluster’s median within each UMAP centroid. For each cluster, the Euclidean distance to the cluster median (UMAP1/UMAP2) was computed. The cutoff defined as median (distance) + threshold × MAD (distance) with a threshold = 2.0 was used for excluding data points.

The corresponding UMAP/clustering outputs for Young and Old datasets were created independently by the above protocol and then merged together for visualization. The same subsets of clusters were maintained for each group: #1 and #2 for Young and #1–#4 for Aged group. UMAP values were then visualized using ggplot2 (features: multivariate normal ellipse; polygon fill; confidence level set to 0.80).

PCA was performed on the track-level mobility feature matrix using the prcomp function in R (R Foundation for Statistical Computing^2^). Prior to PCA, the input features were centered and scaled. The PCA scores (PC coordinates) were extracted for each track point and then plotted. The percentage variance represented by each principal component was evaluated from the Eigen values derived from UMAP coordinates and is shown in the axis labels (PC1: 59.9% and PC2: 20.1%).

### Immunofluorescence

Formalin-fixed paraffin-embedded brains were processed following standard histological procedures. 4-μm sagittal sections were immunostained with antibody for NeuN, IBA1, and P2Y12. DAPI was used to stain nuclei. Fluorescence images were collected with Leica MICA using the 20× dry objective, three images for each cortex. The number of neurons and the percent of neurons in a body-to-body contact with microglia were quantified in representative 1000 × 1000-pixels fields of view selected within each merged image. To calculate mean intensity of IBA1 and P2Y12, three 500 × 500 fields of view in each image of cortices were isolated, IBA1 channel was subjected to a threshold, and a selection for microglial area was created. Then, the mean intensity of IBA1 and P2Y12 was measured within the same selection on the corresponding original channels. The mean intensity of the background was measured and subtracted, and the resulting measurements were averaged for each image.

### Study approval

The study was carried out in strict accordance with the recommendations in the Guide for the Care and Use of Laboratory Animals of the National Institutes of Health. The animal protocol was approved by the Institutional Animal Care and Use Committee of the University of Maryland, Baltimore (Assurance Number: D16-00125; Protocol number: AUP-00000166).

### Statistical analysis

All statistical analysis and graph plotting were performed using GraphPad Prism software, version 10.1.1 for Windows.

## Results

To investigate microglial dynamic behavior, we performed *ex vivo* time-lapse imaging of acute cortical slices prepared from Cx3cr1/EGFP mice. In this model, enhanced green fluorescent protein (EGFP) is expressed under the endogenous Cx3cr1 promoter, restricting labeling to myeloid cells. Two age groups were analyzed: young adult (46–55 days) and aged (605–607 days) mice. Previous studies have shown that morphological, functional, and transcriptional alterations arise in acute brain slices as early as 4 h post-slicing (Ferrucci et al., 2024), and our recent work demonstrated a progressive decline in microglial dynamics beginning 3–4 h post-slicing (Subhramanian et al., 2026). Therefore, all imaging sessions were restricted to a 3-h window following slice preparation. To minimize photobleaching, images were acquired at 3-min intervals.

**FIGURE 1:**
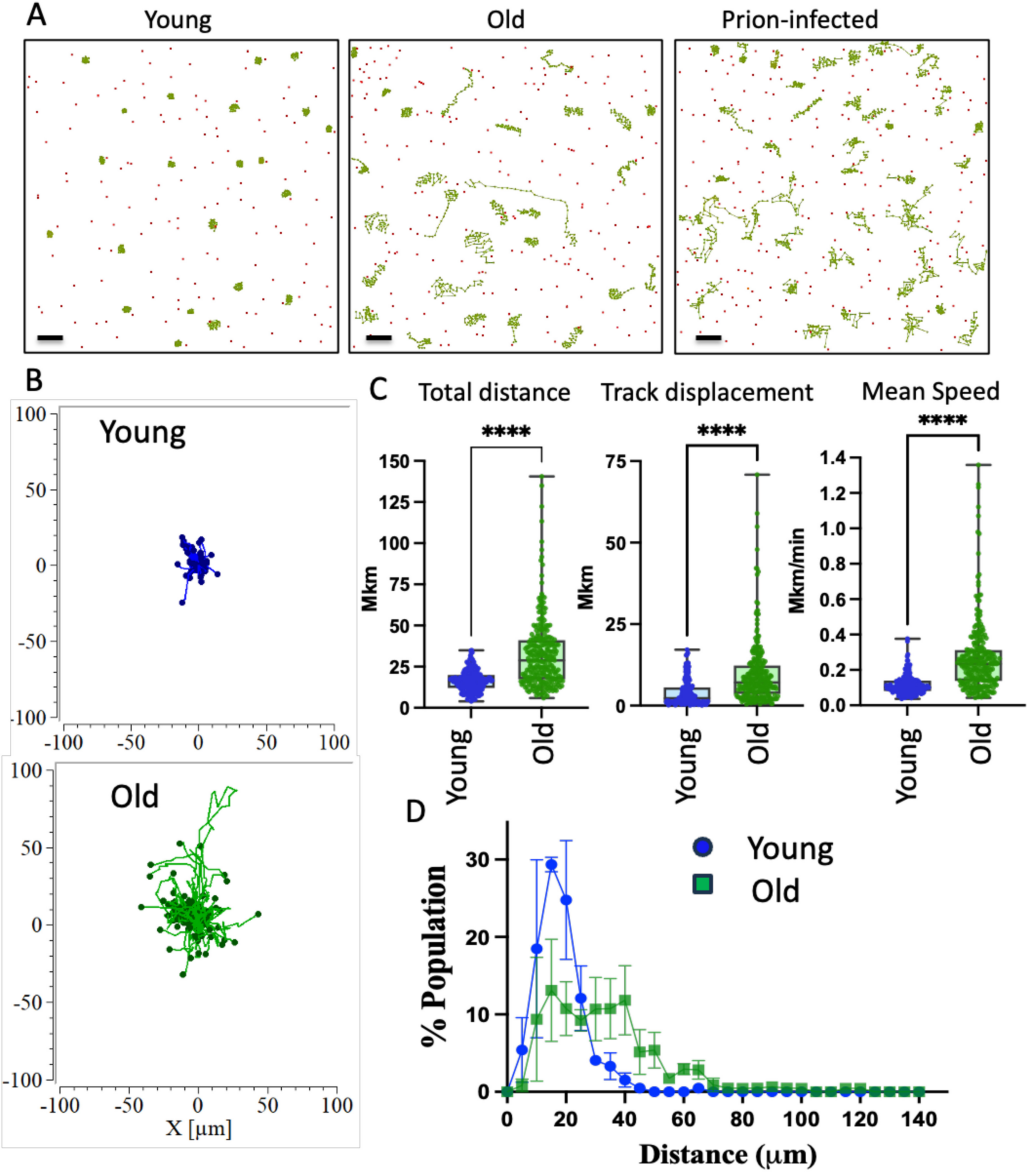
Analysis of microglial mobility in young and aged mice. Acute cerebral cortical slices were prepared from young and aged Cx3cr1/EGFP mice. **(A)** Representative fields of view showing individual cell tracks (green lines) recorded during a 3-h time-lapse imaging session in slices from young, aged, and prion-infected mice. Red dots indicate neuronal positions. **(B)** Rose plots illustrating individual cell trajectories. **(C)** Quantification of total distance traveled, track displacement, and mean speed of individual EGFP^+^ cells over the 3-h imaging period. In box-and-whisker plots, the midline denotes the median, “×” indicates the mean, and box limits represent the 25th and 75th percentiles. *n* = 180 cells (young) and *n* = 270 cells (aged), from three animals per group. ****p < 0.0001 (Mann–Whitney test). **(D)** Histogram showing the distribution of cells according to total distance traveled over 3 h.

In slices from young animals, microglia largely remained confined to defined territories and exhibited minimal somatic displacement (Figures 1A, B). In contrast, microglia in slices from aged animals displayed pronounced somatic mobility, with a subset of cells traversing considerable distances (Figures 1A, B). Consistent with established terminology (Smolders et al., 2019; Subhramanian et al., 2026), we define “mobility” as displacement of the cell soma and “motility” as the movement of microglial processes. Notably, the dynamic behavior of microglia in aged animals resembled that observed in slices from prion-infected mice, where territorial confinement is similarly lost (Figure 1A).

Quantitative analysis of total distance traveled, track displacement (defined as the Euclidean distance between initial and final soma positions), and mean speed confirmed significantly increased mobility in microglia from aged versus young mice (Figure 1C). To determine whether these differences reflect genuine biological differences rather than inter-animal variability, we reanalyzed the data using Superplots, in which cell-level measurements were averaged per animal. These analyses revealed high consistency within each group and minimal inter-animal variability (Supplementary Figures 1A–C).

To further characterize mobility differences, we binned the total distances traveled by individual cells over the 3-h imaging period (Figure 1D). Microglia from aged mice exhibited a pronounced shift toward longer travel distances compared to those from young animals. Moreover, this distribution suggested the presence of distinct subpopulations within the aged group, in contrast to the relatively homogeneous behavior observed in young microglia.

**FIGURE 2:**
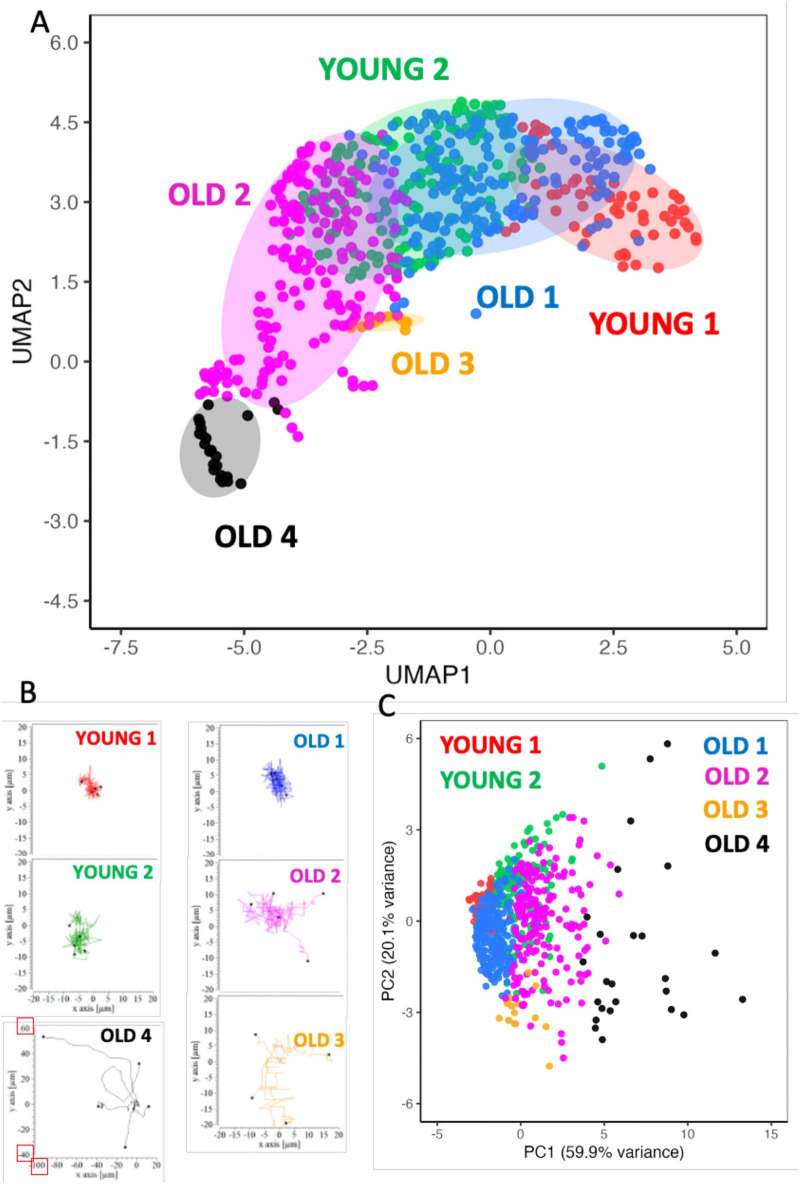
UMAP and PCA analyses of microglia mobility patterns. Clustering of individual cells from young and aged cohorts based on mobility features using UMAP **(A)** and PCA **(C)**. Clusters are color-coded. *n* = 211 cells (young) and *n* = 419 cells (aged), from three animals per group. **(B)** Rose plots of trajectories for cells in clusters 1 and 2 from young animals and clusters 1–4 from aged animals.

**FIGURE 3:**
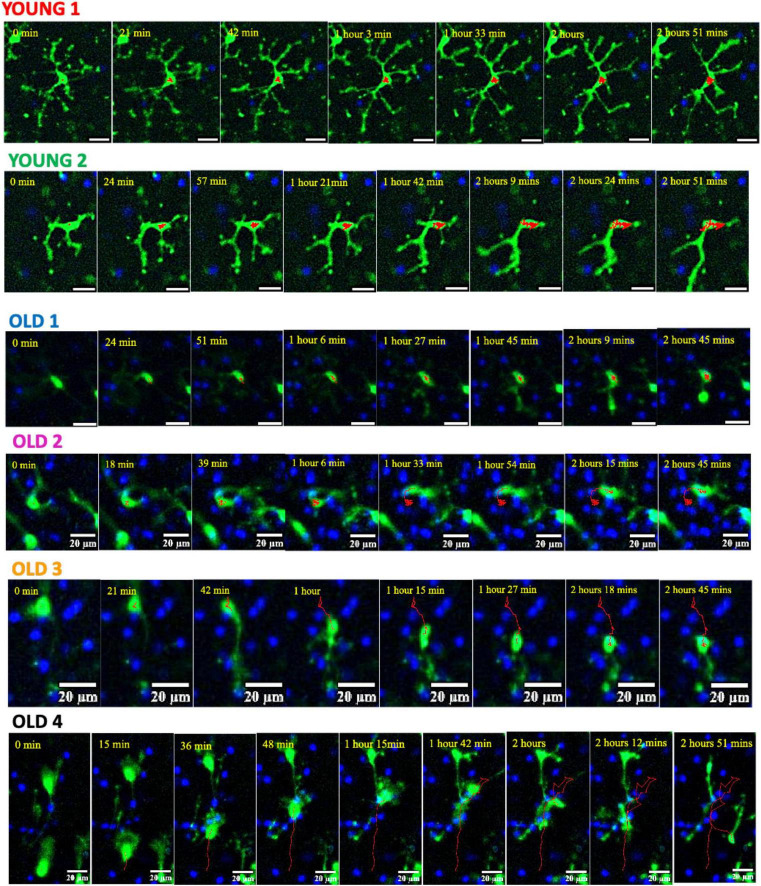
Time-lapse imaging of microglial dynamics in acute cortical slices. Representative cells from clusters 1 and 2 in slices from young animals, and clusters 1–4 in slices from aged animals. Red lines indicate trajectories of individual cells. Time-lapse images were acquired over 3 h. Nuclei were visualized using Hoechst staining. Scale bars = 20 μm.

To resolve this heterogeneity, we performed clustering analyses using Uniform Manifold Approximation and Projection (UMAP) and principal component analysis (PCA), both of which reduce high-dimensional datasets to two-dimensional representations. UMAP identified two clusters in the young group and four clusters in the aged group (Figure 2A). As shown by rose plots (Figure 2B) and time-lapse cell tracking (Figure 3), both clusters of young microglia exhibited limited somatic mobility. These clusters largely overlapped with cluster #1 of the aged group, which also displayed low mobility (Figures 2A,B, 3). Cluster #2 in aged mice partially overlapped with cluster #2 in young mice; however, a substantial fraction of cells within this aged cluster exhibited increased mobility (Figures 2B, 3). Clusters #3 and #4 in aged mice demonstrated the highest mobility, showed no overlap with clusters from young animals, and were also distinct from each other (Figures 2A,B, 3), suggesting the emergence of multiple high-mobility phenotypes with aging. Overall, UMAP analysis reveals an age-associated diversification of microglial dynamic behavior. Independent PCA analysis corroborated these findings, identifying two clusters in young mice and four clusters in aged mice, with clusters #1 and #2 in aged animals overlapping with those observed in the young group (Figure 2C).

Cell tracking further confirmed that in young clusters #1 and #2, microglial somata remained largely stationary while processes exhibited active motility (Figure 3). In contrast, cells in aged cluster #2 displayed short-range somatic displacement, whereas cells in clusters #3 and #4 exhibited long-distance migration (Figure 3).

**FIGURE 4:**
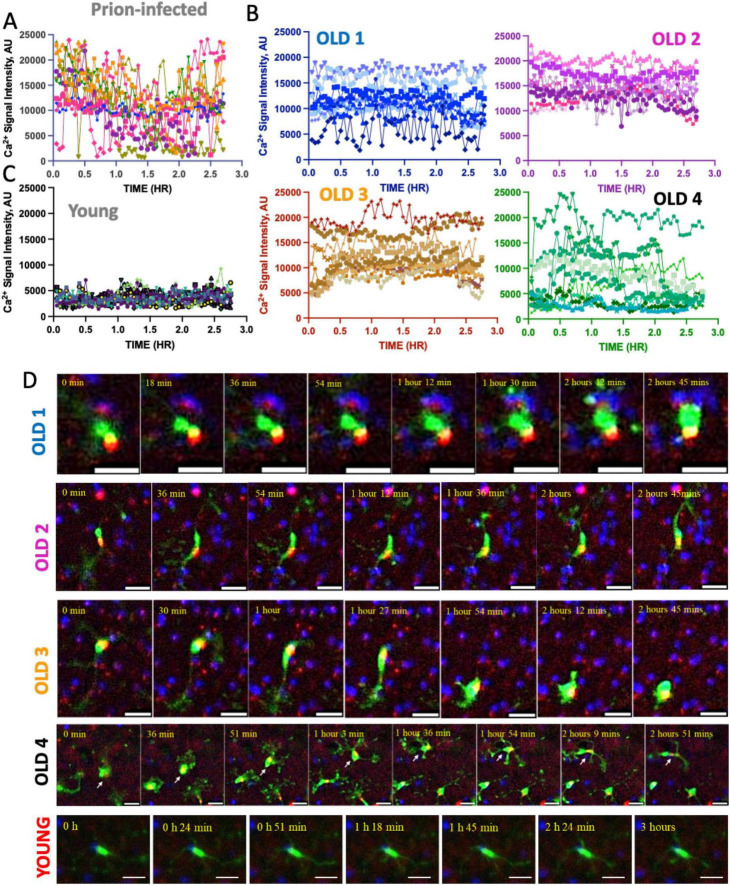
Assessment of Ca^2+^ activity in microglia. **(A,B)** Sustained Ca^2+^ bursts detected using Calbryte-590 AM during 3-h imaging sessions in individual microglial cells from prion-infected animals **(A)** and aged animals (**B**; clusters 1–4). **(C)** Microglia from young animals lack sustained Ca^2+^ bursts. **(D)** Representative time-lapse images showing sustained Ca^2+^ activity (red channel) in clusters 1–4 from aged animals and absence of such activity in young animals. Scale bars: 20 μm.

Microglial activation and migration are known to be regulated by calcium signaling, particularly in response to injury or neuronal damage (Eichhoff et al., 2011; Umpierre et al., 2020, 2024). Consistent with prior studies, we observed sustained calcium bursts in highly mobile microglial subpopulations in slices from prion-infected mice (Figure 4A). Similarly, microglia from aged, animals exhibited prolonged calcium burst (Figures 4B, D). Unexpectedly, in aged mice, these prolonged calcium signals were observed across all microglial clusters, irrespective of their migratory behavior within the 3-h imaging window (Figures 4B, D). In contrast, such sustained calcium activity was absent in young microglia (Figures 4C, D).

In prion-infected brains, microglia acquire a chronically activated, pro-inflammatory phenotype characterized by amoeboid morphology and significant downregulation of homeostatic markers such as P2Y12 (Makarava et al., 2025). These reactive microglia also exhibit soma-to-soma interactions with neurons or neuronal envelopment, distinct from the process-mediated contacts typical of homeostatic microglia (Makarava et al., 2024). To assess whether increased mobility in aged microglia is associated with similar phenotypic changes, we performed co-immunostaining for Iba1, P2Y12, and NeuN. In aged animals, microglia retained a highly ramified morphology, and there was no increase in soma-to-soma contacts with neurons compared to young controls (Supplementary Figure 2). However, we observed a downward trend in P2Y12 expression that approached statistical significance in aged mice, suggesting a modest shift toward an activated phenotype (Supplementary Figure 2).

## Discussion

Little is known about how microglial surveillance strategies adapt to age-related changes. Lifelong exposure to external pathogens and internal stressors, including cellular debris, oxidative stress, and protein aggregates, exerts cumulative effects on microglial function and behavior. It is therefore not surprising that aging is associated with phenotypic alterations in microglia, including features of chronic low-grade inflammation (Holtman et al., 2015; Olah et al., 2018; Hammond et al., 2019; Sankowski et al., 2019). Notably, the overlap between gene sets upregulated in disease-associated microglia (DAM) and subsets of microglia in aged individuals suggests that common transcriptional programs are engaged during both normal aging and neurodegenerative disease. While age-related transcriptomic changes have been well documented, their functional consequences for microglial surveillance behavior remain poorly understood.

Previous work has demonstrated a reduction in the basal motility of microglial processes with aging, suggesting a potential impairment in surveillance mechanisms that rely on process-to-soma purinergic junctions (Hefendehl et al., 2014). A key molecular component of these junctions is the P2Y12 receptor, which mediates microglia-neuron communication under homeostatic conditions (Cserép et al., 2020, 2022). However, P2Y12 gene expression is downregulated with normal aging (Holtman et al., 2015; Galatro et al., 2017). Our findings are consistent with these reports, showing a similar decline at the protein level. How microglia maintain effective neuronal surveillance in aged brains, where P2Y12 expression is diminished despite increased demand for monitoring neuronal health, remains an open question.

In the present study, we observed that a subset of microglia in aged mice lose territorial confinement and exhibit increased somatic mobility. Interestingly, this highly mobile behavior resembles that previously reported in prion-infected brain slices (Subhramanian et al., 2026). Unsupervised clustering revealed the emergence of two highly mobile microglial subpopulations that were absent in young animals. However, in contrast to prion disease, where microglia adopt an amoeboid morphology characteristic of a reactive phenotype, microglia in aged animals retained predominantly ramified morphology. Moreover, we did not detect an increased prevalence of neuronal envelopment in fixed tissue from aged mice. These observations suggest that if direct soma-to-soma microglia-neuron interactions occur during aging, they are likely very short-lived.

The signaling mechanisms underlying enhanced microglial mobility remain poorly defined. Microglial responses to acute injury and infection are known to depend on the purinergic receptor P2Y12, which senses extracellular ATP and ADP and regulates both process extension and directed migration toward sites of tissue damage. However, our recent studies demonstrated that reactive microglia from prion-infected P2Y12-deficient mice retain a highly mobile phenotype comparable to that observed in prion-infected control animals, indicating that elevated microglial mobility is not solely dependent on P2Y12 signaling. The molecular pathways regulating microglial mobility are likely highly redundant. In addition to P2Y12, microglia express several purinergic receptors, including P2Y13, P2Y6, P2X4, and P2X7, which sense extracellular nucleotides and contribute to the regulation of motility, surveillance, and inflammatory responses following tissue injury. Moreover, recent studies have shown that interferon-γ (IFNγ) promotes microglial migration in the adult mouse cortex (Boghozian et al., 2023), raising the possibility that a pro-inflammatory milieu contributes to the heightened intrinsic mobility observed in reactive or primed microglial states. Future studies will be required to determine whether similar mechanisms underlie the emergence of highly mobile microglial subpopulations during normal aging.

Under homeostatic conditions, spontaneous Ca^2+^ transients in microglia are rare, whereas focal neuronal injury induces short Ca^2+^ transients (Brawek et al., 2021). Previous *in vivo* studies have reported increased Ca^2+^ activity beginning in middle-aged mice (9–11 months), characterized by higher frequency, amplitude, and duration of Ca^2+^ events (Olmedillas Del Moral et al., 2019). Consistent with these findings, we observed prolonged Ca^2+^ bursts in microglia from aged animals. Such sustained Ca^2+^ signaling likely reflects a shift toward an altered functional state in response to accumulated age-related stressors. Notably, prolonged Ca^2+^ bursts were observed across microglial clusters irrespective of their mobility profiles, suggesting that even low-mobility microglia in aged animals differ functionally from their counterparts in young brains. Remarkably, the amplitude and duration of Ca^2+^ bursts in aged microglia closely resembled those observed in prion-infected animals, where microglia adopt a chronically reactive phenotype. This parallel further supports the notion that aging is associated with a shift toward a pro-inflammatory microglial state.

Limitations of the present study should be acknowledged. In particular, the potential impact of tissue injury induced by brain slicing on microglial behavior cannot be excluded. However, microglia in slices from young animals exhibited minimal somatic movement following slicing, whereas aged microglia displayed pronounced mobility under the same conditions. This differential response suggests that microglia in aged brains are primed for rapid activation and movement. Importantly, our findings are consistent with prior *in vivo* studies demonstrating an increased proportion of microglia exhibiting spontaneous somatic mobility in aged animals even in the absence of overt injury (Hefendehl et al., 2014).

In summary, this study provides evidence that aging reshapes microglial surveillance by promoting the emergence of highly mobile microglial subpopulations and sustained Ca^2+^ signaling, potentially compensating for the decline in process-mediated monitoring that accompanies aging. These findings raise the possibility that increased soma-mediated mobility represents an adaptive mechanism that helps maintain neuronal surveillance in the aging brain, while simultaneously reflecting a transition toward a primed, disease-associated state. Future studies combining longitudinal *in vivo* imaging, single-cell transcriptomics, and functional manipulation of calcium signaling will be important to determine whether this adaptive response preserves brain homeostasis or instead contributes to the heightened vulnerability of the aged brain to neurodegenerative disorders.

## Data Availability

The original contributions presented in this study are included in this article/Supplementary material, further inquiries can be directed to the corresponding author.

## Footnotes

1
https://www.r-project.org/

2
https://www.r-project.org

## References

[B1] Boghozian R. Sharma S. Narayana K. Cheema M. Brown C. E. (2023). Sex and interferon gamma signaling regulate microglia migration in the adult mouse cortex *in vivo*. *Proc. Natl. Acad. Sci. U. S. A.* 120:e2302892120. 10.1073/pnas.2302892120 37428916 PMC10629543

[B2] Brawek B. Skok M. Garaschuk O. (2021). Changing functional signatures of microglia along the axis of brain aging. *Int. J. Mol. Sci.* 22:1091. 10.3390/ijms22031091 33499206 PMC7865559

[B3] Butovsky O. Weiner H. L. (2018). Microglial signatures and their role in health and disease. *Nat. Rev. Neurosci.* 19 622–635. 10.1038/s41583-018-0057-5 30206328 PMC7255106

[B4] Cserép C. Pósfai B. Lénárt N. Fekete R. László Z. I. Lele Z. et al. (2020). Microglia monitor and protect neuronal function through specialized somatic purinergic junctions. *Science* 367 528–537. 10.1126/science.aax6752 31831638

[B5] Cserép C. Schwarcz A. D. Pósfai B. László Z. I. Kellermayer A. Környei Z. et al. (2022). Microglial control of neuronal development via somatic purinergic junctions. *Cell Rep.* 40:111369. 10.1016/j.celrep.2022.111369 36130488 PMC9513806

[B6] Damani M. R. Zhao L. Fontainhas A. M. Amaral J. Fariss R. N. Wong W. T. (2011). Age-related alterations in the dynamic behavior of microglia. *Aging Cell* 10 263–276. 10.1111/j.1474-9726.2010.00660.x 21108733 PMC3056927

[B7] Eichhoff G. Brawek B. Garaschuk O. (2011). Microglial calcium signal acts as a rapid sensor of single neuron damage *in vivo*. *Biochim. Biophys. Acta* 1813 1014–1024. 10.1016/j.bbamcr.2010.10.018 21056596

[B8] Ferrucci L. Basilico B. Reverte I. Pagani F. Scaringi G. Cordella F. et al. (2024). Time-dependent phenotypical changes of microglia drive alterations in hippocampal synaptic transmission in acute slices. *Front. Cell. Neurosci.* 18:1456974. 10.3389/fncel.2024.1456974 39619853 PMC11604457

[B9] Galatro T. F. Holtman I. R. Lerario A. M. Vainchtein I. D. Brouwer N. Sola P. R. et al. (2017). Transcriptomic analysis of purified human cortical microglia reveals age-associated changes. *Nat. Neurosci.* 20 1162–1171. 10.1038/nn.4597 28671693

[B10] Hammond T. R. Dufort C. Dissing-Olesen L. Giera S. Young A. Wysoker A. et al. (2019). Single-cell RNA sequencing of microglia throughout the mouse lifespan and in the injured brain reveals complex cell-state changes. *Immunity* 50 253–271.e6. 10.1016/j.immuni.2018.11.004 30471926 PMC6655561

[B11] Hefendehl J. K. Neher J. J. Sühs R. B. Kohsaka S. Skodras A. Jucker M. (2014). Homeostatic and injury-induced microglia behavior in the aging brain. *Aging Cell* 13 60–69. 10.1111/acel.12149 23953759 PMC4326865

[B12] Holtman I. R. Raj D. D. Miller J. A. Schaafsma W. Yin Z. Brouwer N. et al. (2015). Induction of a common microglia gene expression signature by aging and neurodegenerative conditions: A co-expression meta-analysis. *Acta Neuropathol. Commun.* 3:31. 10.1186/s40478-015-0203-5 26001565 PMC4489356

[B13] Keren-Shaul H. Spinrad A. Weiner A. Matcovitch-Natan O. Dvir-Szternfeld R. Ulland T. K. et al. (2017). A unique microglia type associated with restricting development of Alzheimer’s disease. *Cell* 169 1276–1290.e17. 10.1016/j.cell.2017.05.018 28602351

[B14] Krasemann S. Madore C. Cialic R. Baufeld C. Calcagno N. El Fatimy R. et al. (2017). The TREM2-APOE pathway drives the transcriptional phenotype of dysfunctional microglia in neurodegenerative diseases. *Immunity* 47 566–581.e9. 10.1016/j.immuni.2017.08.008 28930663 PMC5719893

[B15] Makarava N. Chang J. C. Molesworth K. Baskakov I. V. (2020). Region-specific glial homeostatic signature in prion diseases is replaced by a uniform neuroinflammation signature, common for brain regions and prion strains with different cell tropism. *Neurobiol. Dis.* 137:104783. 10.1016/j.nbd.2020.104783 32001329 PMC7052953

[B16] Makarava N. Mychko O. Chang J. C. Molesworth K. Baskakov I. V. (2021). The degree of astrocyte activation is predictive of the incubation time to prion disease. *Acta Neuropathol. Commun.* 9:87. 10.1186/s40478-021-01192-9 33980286 PMC8114720

[B17] Makarava N. Safadi T. Bocharova O. Mychko O. Pandit N. P. Molesworth K. et al. (2024). Reactive microglia partially envelop viable neurons in prion diseases. *J. Clin. Invest.* 134:e181169. 10.1172/JCI181169 39361421 PMC11601909

[B18] Makarava N. Safadi T. Bocharova O. Mychko O. Pandit N. P. Molesworth K. et al. (2025). Knockout of P2Y12 receptor facilitates neuronal envelopment by reactive microglia and accelerates prion disease. *J. Neuroinflammation* 22:210. 10.1186/s12974-025-03542-z 40887594 PMC12400595

[B19] Nebeling F. C. Poll S. Justus L. C. Steffen J. Keppler K. Mittag M. et al. (2023). Microglial motility is modulated by neuronal activity and correlates with dendritic spine plasticity in the hippocampus of awake mice. *Elife* 12:e83176. 10.7554/eLife.83176 36749020 PMC9946443

[B20] Olah M. Patrick E. Villani A. C. Xu J. White C. C. Ryan K. J. et al. (2018). A transcriptomic atlas of aged human microglia. *Nat. Commun.* 9:539. 10.1038/s41467-018-02926-5 29416036 PMC5803269

[B21] Olmedillas Del Moral M. Asavapanumas N. Uzcátegui N. L. Garaschuk O. (2019). Healthy brain aging modifies microglial calcium signaling *in vivo*. *Int. J. Mol. Sci.* 20:589. 10.3390/ijms20030589 30704036 PMC6386999

[B22] Rotterman T. M. Alvarez F. J. (2020). Microglia dynamics and interactions with motoneurons axotomized after nerve injuries revealed by two-photon imaging. *Sci. Rep.* 10:8648. 10.1038/s41598-020-65363-9 32457369 PMC7250868

[B23] Sankowski R. Böttcher C. Masuda T. Geirsdottir L. Sagar, Sindram E. et al. (2019). Mapping microglia states in the human brain through the integration of high-dimensional techniques. *Nat. Neurosci.* 22 2098–2110. 10.1038/s41593-019-0532-y 31740814

[B24] Sierra A. Gottfried-Blackmore A. C. McEwen B. S. Bulloch K. (2007). Microglia derived from aging mice exhibit an altered inflammatory profile. *Glia* 55 412–424. 10.1002/glia.20468 17203473

[B25] Smolders S. M. Kessels S. Vangansewinkel T. Rigo J. M. Legendre P. Brône B. (2019). Microglia: Brain cells on the move. *Prog. Neurobiol.* 178:101612. 10.1016/j.pneurobio.2019.04.001 30954517

[B26] Subhramanian S. Bocharova O. Makarava N. Safadi T. Baskakov I. V. (2026). Dissecting surveying behavior of reactive microglia under chronic neurodegeneration. *Elife* 14:RP107650. 10.7554/eLife.107650 41504596 PMC12782555

[B27] Umpierre A. D. Bystrom L. L. Ying Y. Liu Y. U. Worrell G. Wu L.-J. (2020). Microglial calcium signaling is attuned to neuronal activity in awake mice. *Elife* 9:e56502. 10.7554/eLife.56502 32716294 PMC7402678

[B28] Umpierre A. D. Li B. Ayasoufi K. Simon W. L. Zhao S. Xie M. et al. (2024). Microglial P2Y6 calcium signaling promotes phagocytosis and shapes neuroimmune responses in epileptogenesis. *Neuron* 112:1959–1977.e10. 10.1016/j.neuron.2024.03.017 38614103 PMC11189754

