# Supplementary material for: Age-associated microglial heterogeneity includes emergence of mobile microglial states
Source: Front Aging Neurosci. 2026 Jun 30;18:1847223. doi: 10.3389/fnagi.2026.1847223 (PMC13365027; doi:10.3389/fnagi.2026.1847223)
Supplement: Supplementary file 1 [file Data_Sheet_1.DOCX]

**Supplemental Data**

**A shift in microglial surveillance strategy during brain aging**

**Sunitha Subhramanian^1^, Olga Bocharova^1^, Olga Mychko^1^, Natallia Makarava^1^, Ilia V. Baskakov^1*^**

^1^ Department of Neurobiology, University of Maryland School of Medicine, Baltimore, Maryland, USA

**
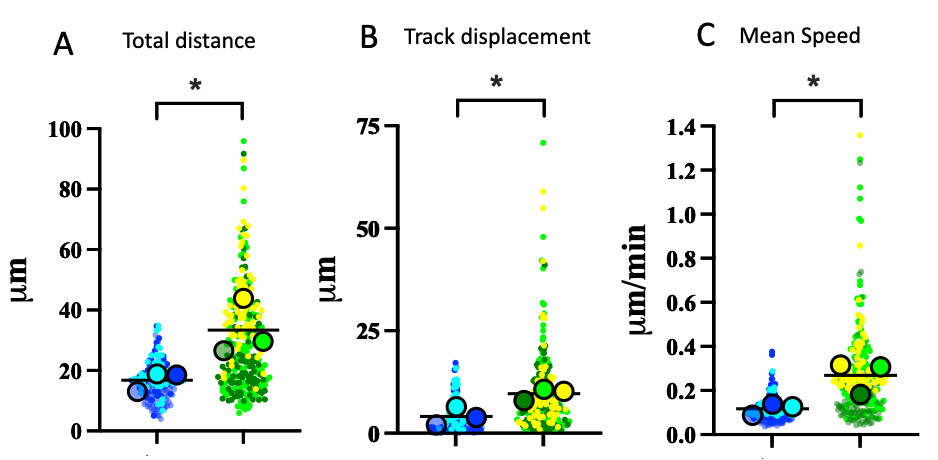
**

**Figure S1. Inter-animal variation in microglia mobility.** Analysis of total distance (**A**), track displacement (**B**), and mean speed (**C**) of individual cells monitored over a 3-hour period in cerebral cortical slices from young and old mice. In Superplots, colors denote individual animals, dots represent single cells, circles show average value per animal, black lines indicate group means calculated from biological replicates (animals). N=3 animals per group. Significance was determined by ordinary one-way ANOVA: *p<0.05.

**
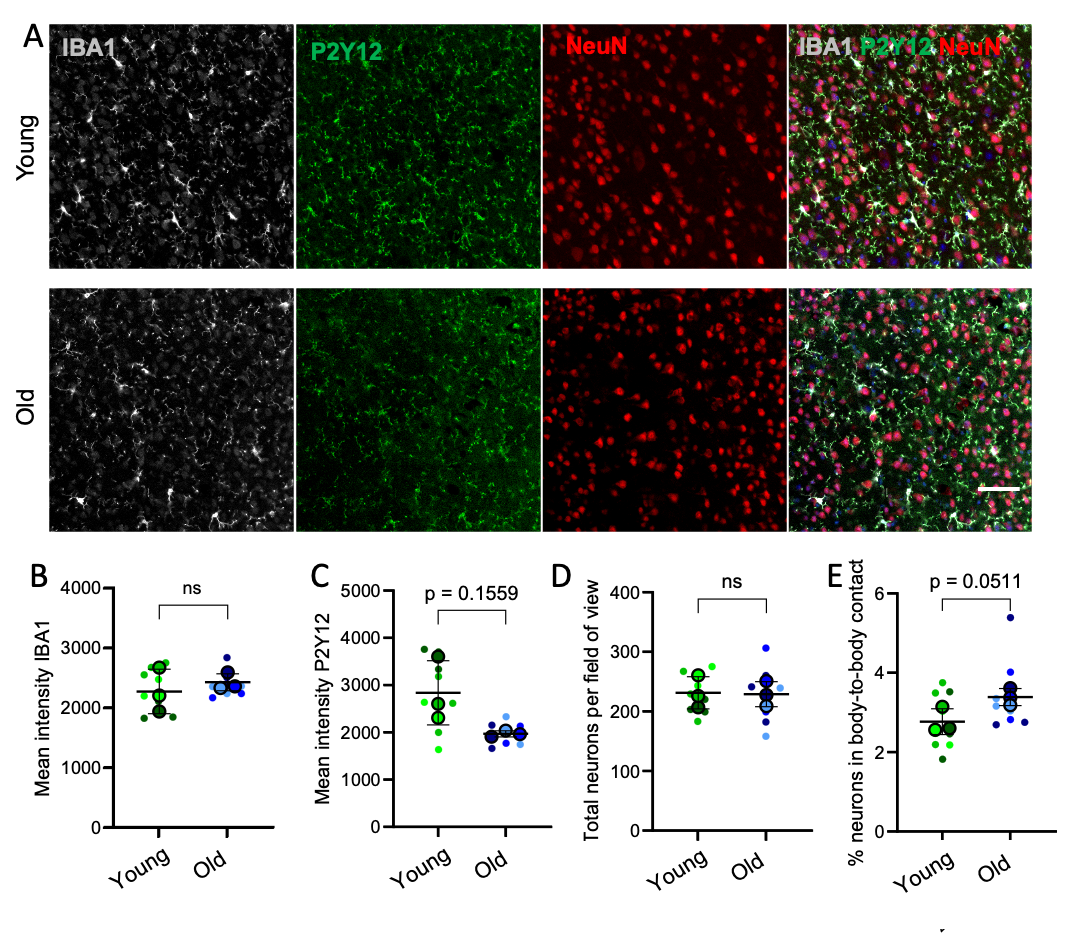
**

**Figure S2. Analysis of P2Y12 expression and microglia-neuron soma contacts.** (**A**) Co-immunostaining of cortical sections from young and aged animals using anti-IBA1 (pseudo-white), anti-P2Y12 (green), and anti-NeuN (red) antibodies. (**B–E**) Quantification of mean IBA1 intensity (**B**), mean P2Y12 intensity (**C**), number of neurons per field of view (**D**), and percentage of neurons in soma-to-soma contact with microglia (**E**). In Superplots, colors represent individual animals; dots correspond to individual images; and circles indicate per-animal averages. Statistical comparisons were performed using an unpaired Student’s t-test, except for P2Y12 intensity, which was analyzed using Welch’s t-test. ns, not significant. N = 3 animals per group, with three fields of view per animal. Scale bar: 50 μm.
